# PHA Production and PHA Synthases of the Halophilic Bacterium *Halomonas* sp. SF2003

**DOI:** 10.3390/bioengineering7010029

**Published:** 2020-03-20

**Authors:** Tatiana Thomas, Kumar Sudesh, Alexis Bazire, Anne Elain, Hua Tiang Tan, Hui Lim, Stéphane Bruzaud

**Affiliations:** 1 Institut de Recherche Dupuy de Lôme (IRDL), Université de Bretagne Sud (UBS), EA 3884 Lorient, France; stephane.bruzaud@univ-ubs.fr; 2 Institut de Recherche Dupuy de Lôme (IRDL), Université de Bretagne Sud (UBS), 56300 Pontivy, France; anne.elain@univ-ubs.fr; 3 School of Biological Sciences, Universiti Sains Malaysia (USM), Penang 11800, Malaysia; ksudesh@usm.my (K.S.); tiang93@hotmail.com (H.T.T.); lhlim1993@gmail.com (H.L.); 4 Laboratoire de Biotechnologie et Chimie Marines (LBCM), IUEM, Université de Bretagne-Sud (UBS), EA 3884 Lorient, France; alexis.bazire@univ-ubs.fr

**Keywords:** halophilic bacteria, polyhydroxyalkanoates (PHAs), PHA synthases

## Abstract

Among the different tools which can be studied and managed to tailor-make polyhydroxyalkanoates (PHAs) and enhance their production, bacterial strain and carbon substrates are essential. The assimilation of carbon sources is dependent on bacterial strain’s metabolism and consequently cannot be dissociated. Both must wisely be studied and well selected to ensure the highest production yield of PHAs. *Halomonas* sp. SF2003 is a marine bacterium already identified as a PHA-producing strain and especially of poly-3-hydroxybutyrate (P-3HB) and poly-3-hydroxybutyrate-*co*-3-hydroxyvalerate (P-3HB-*co*-3HV). Previous studies have identified different genes potentially involved in PHA production by *Halomonas* sp. SF2003, including two *phaC* genes with atypical characteristics, *phaC1* and *phaC2*. At the same time, an interesting adaptability of the strain in front of various growth conditions was highlighted, making it a good candidate for biotechnological applications. To continue the characterization of *Halomonas* sp. SF2003, the screening of carbon substrates exploitable for PHA production was performed as well as production tests. Additionally, the functionality of both PHA synthases PhaC1 and PhaC2 was investigated, with an *in silico* study and the production of transformant strains, in order to confirm and to understand the role of each one on PHA production. The results of this study confirm the adaptability of the strain and its ability to exploit various carbon substrates, in pure or mixed form, for PHA production. Individual expression of PhaC1 and PhaC2 synthases in a non-PHA-producing strain, *Cupriavidus necator* H16 PHB¯4 (DSM 541), allows obtaining PHA production, demonstrating at the same time, functionality and differences between both PHA synthases. All the results of this study confirm the biotechnological interest in *Halomonas* sp. SF2003.

## 1. Introduction

Polyhydroxyalkanoates (PHAs) are valuable bio-based and biodegradable polymers produced by numerous bacterial species [1,2]. Their properties are close to those of conventional petroleum-based plastics; therefore, in addition to their biocompatibility, they are considered to be materials with high potential [3]. Actually, they can be used in various fields ranging from packaging [2] to biomedical applications [1,4], but one of the main obstacles to their commercialization and exploitation is the overall cost of production. Currently, several tools can be managed to reduce the final cost of PHA production, including the characterization of selected microorganisms coupled with the optimal selection of carbon substrates [5,6]. Indeed, a better understanding of strain metabolisms and response in front of different growth and/or production conditions participate to tailor-make PHA and enhance production yield. To date, there is an important diversity of carbon sources (monomers) that can be exploited for PHA synthesis, and as a result, a wide range of PHAs which can be synthesized [6]. PHA properties are closely linked to their bacterial producer strain, carbon substrates, and production mode [6,7], meaning that an accurate study of each parameter is required. On the other hand, the PHA production cost is still limiting their more widespread use. Over the last decade, research has notably focused on the use of low-value substrates like industrial co-products (from agri-food, waste treatment, or biodiesel industry) [6,8,9] as they can represent up to 50% of the production cost. With these carbon substrates, perfect control of PHA’s structure, molecular weight and properties could be difficult. Therefore, complete studies of carbon substrates utilization and PHA synthesis are required to soundly select the most adapted carbon sources, whether it is pure carbohydrates or co-products. Another way to reduce production costs is to study the strain genome using bioinformatics and genetic engineering. These tools are also exploiting for expression of PHA synthesis operon in non-producing strains exposing less restrictive growth and production conditions [5,10].

*Halomonas* sp. SF2003 is a halophilic bacterium identified as a PHA-producing strain [11]. Previous studies have shown its capacity to produce polymer up to 78 wt% of cell dry weight (CDW), using conventional carbon sources but also carbonaceous by-products from food wastes [12]. Bioinformatics and phenotypic studies of *Halomonas* sp. SF2003 have demonstrated its versatility under various atypical growth conditions making it an adaptable bacterium. Additionally, genomic annotation also allows identifying various metabolic pathways directly involved, or not, in the synthesis of PHA, which can be studied for a stronger understanding of *Halomonas* sp. SF2003 PHA metabolism. Our previous study highlighted atypical characteristics and organization of PHA biosynthesis genes (*phaA*, *phaB*, *phaC1*, *phaC2,* and *phaR*) [13]. Regarding its original properties, *Halomonas* sp. SF2003 is an excellent candidate for the innovative development of biotechnological production of PHA.

The objectives of this work were to go further into the unraveling/understanding of PHA biosynthesis capability and metabolism of *Halomonas* sp. SF2003 and to identify potential carbon substrates, and in later stage, potential industrial co-products, which can be exploited for PHA production. Our work will also contribute to better understand the functionality of both PHA synthases of *Halomonas* sp. SF2003 in order to later optimize its PHA production.

## 2. Materials and Methods

### 2.1. Bacterial Strains and Media

#### 2.1.1. Bacterial Strains

All the bacterial strains used in this study have been furnished by Research Institute Dupuy de Lôme (RIDL, University of South Brittany) collections or have been purchased to the Deutsche Sammlung von Mikroorganismen und Zellkulturen (DSMZ) collection (Table 1).

#### 2.1.2. Growth Media

*Halomonas* sp. SF2003 is cultivated in Zobell medium (Bacto Tryptone, (Difco, BD, Göteborg, Sweden) 4 g/L, Yeast Extract (Fisher BioReagents, Pittsburgh, PA, USA) 1 g/L, sea salts (Aquarium systems, Instant Ocean, Blacksburg, VA, USA) 30 g/L, pH 7.5), with an orbital agitation of 200 rpm, at 30 °C. The medium is complemented with glucose (Labogros), at 10 g/L, for pre-cultures dedicated to PHA productions.

*Cupriavidus necator* H16, *C. necator* PHB¯4, PHB¯4/pBBR1-Pro_Cn_-*phaC1* and PHB¯4/pBBR1-Pro_Cn_-*phaC2* are cultivated in nutrient-rich medium (NR medium) (Meat extract (Biokar Diagnostics, Allonne, France) 10 g/L, Yeast extract (Fisher BioReagents, USA) 2 g/L, Peptone from gelatin, enzymatic digest (Sigma-Aldrich, St. Louis, MO, USA) 10 g/L, pH (7)), with an orbital agitation of 200 rpm, at 30 °C.

The transformant strains PHB¯4/pBBR1-Pro_Cn_-*phaC1* and PHB¯4/pBBR1-Pro_Cn_-*phaC2* were selected on Simmons citrate agar plates (Thermo Scientific™, Illkirch–Graffenstaden, France), prepared following the manufacturer instructions. For the transformant strains PHB¯4/pBBR1-Pro_Cn_-*phaC1* and PHB¯4/pBBR1-Pro_Cn_-*phaC2*, the media were complemented with kanamycin (Km, Gibco, Waltham, MA, USA), at 50 µg/mL.

#### 2.1.3. Production Media

The PHA productions were performed using a two-steps protocol. Biomass accumulation was performed in Reference 1 medium (carbon source 10 g/L, Bacto Tryptone (Difco, BD, Sweden) 1 g/L, Yeast extract (Fisher BioReagents, USA) 0.5 g/L, sea salts (Aquarium systems, Instant Ocean, USA) 11 g/L, pH (7.5) and C/N ratio 24.6), and PHA production was performed in Reference 2 medium (carbon source 20 g/L, Yeast extract (Fisher BioReagents, USA) 0.4 g/L, sea salts (Aquarium systems, Instant Ocean, USA) 11 g/L, pH 7.0 and C/N ratio 187.2). Reference 2 medium was also employed for the screening of carbon sources usable for PHA production. Nile Red agar plates have been prepared by adding agar powder (15 g/L, Fisher BioReagents, USA) and filtered Nile Red (0.5% (w/v), Sigma-Aldrich, USA) to Reference 2 medium. For the transformant strains PHB¯4/pBBR1-Pro_Cn_-*phaC1* and PHB¯4/pBBR1-Pro_Cn_-*phaC2*, the media were complemented with kanamycin (Km, Gibco, USA), at 50 µg/mL.

### 2.2. In Silico Study of PHA Synthase of Halomonas sp. SF2003 PhaC1 and PhaC2

In silico analysis of PhaC1 and PhaC2 synthases of *Halomonas* sp. SF2003 was performed by confronting amino acids sequences of PHA synthases from several PHA-producing strains (all the sequences tested are available on the National Center for Biotechnology Information (NCBI) database, and accession numbers are available in the Supplementary data S1: Accession numbers of PhaC amino acids sequence). The identification of lipase box-like sequences was conducted using BioEdit software using the ClustalW Multiple alignment tool.

### 2.3. Cloning of Halomonas sp. SF2003 phaC1 and phaC2 Genes

**Figure 1:**
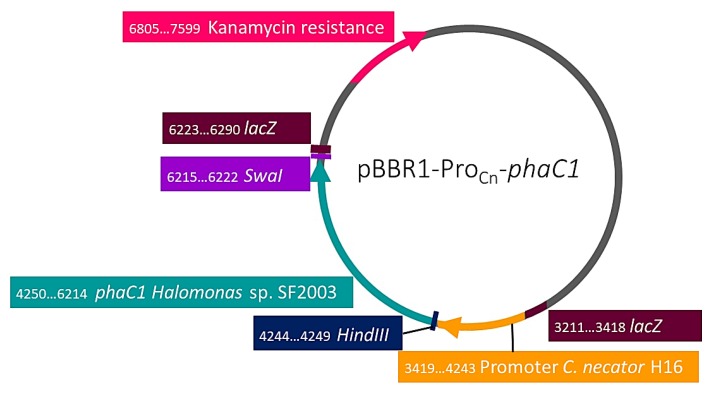
pBBR1-Pro_Cn_-*phaC1* plasmid map.

**Figure 2:**
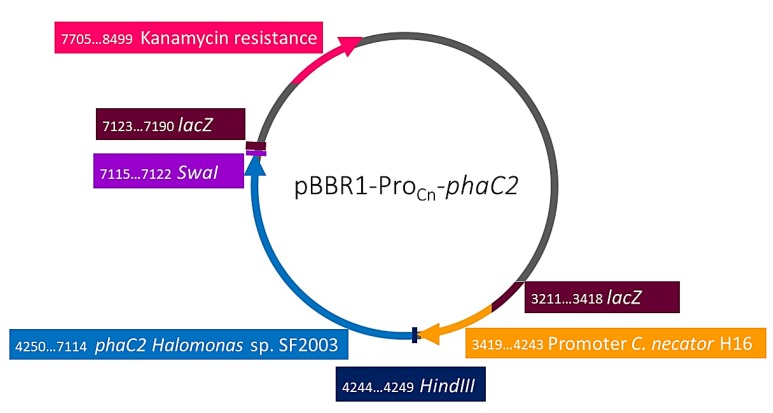
pBBR1-Pro_Cn_-*phaC2* plasmid map.

To evaluate the activity of PHA synthases PhaC1 and PhaC2 of *Halomonas* sp. SF2003, *phaC1*, and *phaC2* genes were cloned. Genomic DNA of *Halomonas* sp. SF2003 overnight cultures in Zobell medium was extracted using QIAamp DNA Mini Kit (Qiagen©, Hilden, Germany). The primers PhaC1-F (5’-AGTAAGCTTAGGAGGAGGCGCATGCAGTCGCCAGCCCA-3’), PhaC1-R (5’-AGTAGCATTTAAATTCAG-GTTTGCTTCACGTAGGTG-3’), PhaC2-F (5’-AGTAAGCTTAGGAGGAGGCGCATGGACTCAGCCCAGCA-3’) and PhaC2-R (5’-AGTAGCATTTAAATTCAACTCTTGTCGCTATCCTTGG-3’) were designed based on the nucleotide sequence of *Halomonas* sp. SF2003 and using A plasmid Editor software (ApE). The PCR reactions were performed using KAPA HiFi HotStart Ready Mix PCR Kit (Kapa Biosystems, Wilmington, MA, USA) and an MJ Mini Thermal Cycler (BioRad, Hercules, CA, USA) according to the manufacturer’s instructions and applying the following parameters: initial denaturation for 3 min at 98 °C, denaturation for 20 s at 98 °C, primer annealing for 15 s at 54 °C, elongation for 40 s at 72 °C, and final extension for 2 min at 72 °C. The denaturation, primer annealing and extension steps were repeated 30 times. Each amplicon has been digested using *Swa*I and *Hind*III enzymes before to be ligated with the pBBR1-Pro_Cn_ plasmid using DNA ligation kit (TaKaRa Bio Inc., Kyoto, Japan), with an insert/vector ratio of 3:1 and following the manufacturer’s instructions, to obtain pBBR1-Pro_Cn_-*phaC1* and pBBR1-Pro_Cn_-*phaC2* plasmids (Figure 1 and Figure 2). The resulting plasmids were then used to be cloned into *E. cloni*^®^ 10G cells (Lucigen Corporation, Middleton, WI, USA) by thermal shock of 45 s at 42 °C. Transformant cells were selected on Luria Bertani (LB) agar plates complemented with kanamycin (50 µg/mL) and after control PCR. Plasmid extraction was performed using the GeneJET Plasmid Miniprep Kit (Thermo Scientific™, France) and following the manufacturer’s instructions. The extracted plasmids were used to transform *E. coli* S17-1 competent cells by a thermal shock of 45 s at 42 °C; transformant cells were selected on LB agar plates complemented with kanamycin (50 µg/mL). A bacterial glycerol stock of the plasmids was with a final glycerol concentration of 25% and stocked at −80 °C. Then, transconjugation between *E. coli* S17-1 cells, harboring pBBR1-Pro_Cn_-*phaC1* or pBBR1-Pro_Cn_-*phaC2* plasmids, and *C. necator* PHB¯4 was performed by mixing liquid cultures of each strain before to inoculate NR agar plate and incubate it for 8 h at 30 °C. After the incubation time, colonies were picked up and used to prepare an NR medium suspension. Simmons citrate agar plates complemented with kanamycin were inoculated and incubated for two days at 30 °C. Blue colonies were picked up and used to perform control PCR using EconoTaq Master Mix (Lucigen Corporation, USA) and following the manufacturer’s instructions, as the same time than subculture on LB agar plates complemented with kanamycin (50 µg/mL). Plasmid extraction was performed using the GeneJET Plasmid Miniprep Kit (Thermo Scientific™, France) and following manufacturer instructions. DNA sequencing was done by 1st BASE Sdn. Bhd. (Malaysia).

### 2.4. Screening for Carbon Sources

A total of eight pure carbohydrates: fructose, galactose, glucose, maltose, mannose, melibiose, rhamnose and sucrose; and seven organic acids: dodecanoic acid, heptanoic acid, hexanoic acid, levulinic acid, malic acid, palmitic acid, and trans-2-pentenoic acid in mixture with fructose, galactose or glucose; were tested for PHA accumulation. These carbon sources were selected depending on their origin and were tested for PHA accumulation using Nile Red agar plates technique [11]. All the reagents were purchased from Sigma-Aldrich (St. Louis, MO, USA) or Thermo Fisher Scientific (Illkirch-Graffenstaden, France).

Overnight pre-cultures of *Halomonas* sp. SF2003, *C. necator* H16, *C. necator* PHB¯4, PHB¯4/pBBR1-Pro_Cn_-*phaC1*, and PHB¯4/pBBR1-Pro_Cn_-*phaC2* were used to screen carbon sources assimilation on Nile Red agar plates. After three days of incubation at 30 °C, bacterial growth and fluorescence were checked under white and UV light.

### 2.5. PHA Production

The PHA productions were composed of three steps: pre-culture, biomass accumulation, and polymer production.

Pre-cultures were performed for 7 h at 30 °C in the Zobell medium complemented with 10 g/L of glucose for *Halomonas* sp. SF2003 and in NR medium for *C. necator* H16 (DSM 428) or NR medium complemented with kanamycin (50 µg/mL) for PHB¯4/pBBR1-Pro_Cn_-*phaC1* and PHB¯4/pBBR1-Pro_Cn_-*phaC2*.

For the biomass accumulation step, Reference 1 medium was inoculated at 10% (v/v) with pre-cultures, and incubated at 30 °C for 17 h with an orbital shaking of 200 rpm. Biomass accumulation was monitored by OD_600nm_ measurement. Once the maximum biomass was reached, cultures were stopped, harvested, and centrifuged at 7500 rpm for 10 min at 4 °C. Then, cells were washed twice with saline water (sea salts (Aquarium systems, Instant Ocean, USA) 11 g/L) and centrifuged at 7500 rpm for 10 min at 4 °C before resuspension in a minimal volume of saline water.

The PHA production step was initiated by transfer of cell pellet of the previous step into Reference 2 medium. The cultivation was performed for 72 h at 30 °C with an orbital shaking of 200 rpm. At the end of the step, the bacterial culture was harvested and centrifuged. Cell pellets were washed with distilled water before freezing at −80 °C and freeze-drying for 48 h.

For productions in shake flasks, production volumes were designed to only use a fifth or a quarter of the maximum volumes of shake flasks in order to conserve a sufficient contact surface with air, allowing oxygenation.

### 2.6. PHA Extraction

Lyophilized cells were manually ground before performing PHA extraction using chloroform (25 mL of solvent per g of CDW) at 60 °C for 15–16 h. After the dissolution of the PHA in chloroform, distilled water was added, 1/3 of the total volume of chloroform, and the suspension was vigorously agitated before centrifugation at 5000 rpm for 7 min. Then, the organic phase was recovered using a sterile syringe and filtered using glass cotton to remove cellular debris. Then, the solvent was evaporated, and PHA films were solvent-casted in a glass Petri dish, at room temperature, until a constant weight obtained. The PHA content was determined as the PHA to cell dry weight (CDW) percent ratio [12,16].

## 3. Results

### 3.1. In Silico Study of PHA Synthases PhaC1 and PhaC2 of Halomonas sp. SF2003

In the previous work, the whole genome of *Halomonas* sp. SF2003 was sequenced and annotated, leading to the identification of two genes potentially encoding two distinct PHA synthase proteins PhaC1 and PhaC2; belonging to class I (based on gene organization and biosynthesized PHA) [13]. To further characterize this first analysis, the consensus lipase box-like sequence of both PHA synthases have been studied.

**Figure 3:**
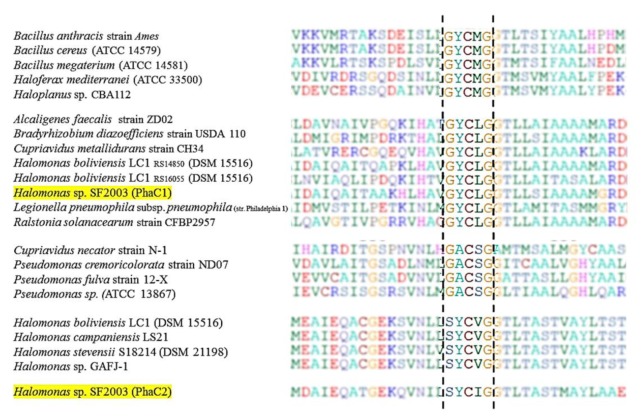
Multiple alignments of partial amino acid sequences of PHA synthase exposing lipase box-like patterns from different bacterial species. All the sequences are available on the National Center for Biotechnology Information (NCBI) database. Highlighted sequences correspond to PHA synthases PhaC1 and PhaC2 of *Halomonas* sp. SF2003.

Amino acid sequences of PHA synthases, PhaC1 and PhaC2 of *Halomonas* sp. SF2003 have been analyzed and allowed the identification of two distinct lipase box-like patterns in both enzymes, beginning at position 384 for PhaC1 and position 343 for PhaC2. In the PhaC1 sequence, the pattern is composed of Glycine-Tyrosine-Cysteine-Leucine-Glycine (G-Y-C-L-G), and pattern in PhaC2 is Serine-Tyrosine-Cysteine-Isoleucine-Glycine (S-Y-C-I-G) (Figure 3). Results obtained for PHA synthases of *Halomonas* sp. SF2003 still demonstrated the distinction of both enzymes, additionally to their size and location in the genome [13]. Indeed, two different patterns have been reported: G-Y-C-L-G for PhaC1 and S-Y-C-I-G for PhaC2. The existence of different PHA synthase enzymes in the same bacterial strain has already been observed, as well as several lipase box-like sequences, like for *Halomonas boliviensis* LC1 (DSM 15516). This strain has several PHA synthases in which different lipase box-like pattern have been detected (Figure 3). Additionally, to this difference of pattern in PhaC box consensus sequences, analysis of amino acid sequences framing these active sites suggests a difference in the final structure of proteins.

### 3.2. Screening of Carbon Substrates for PHA Production by Halomonas sp. SF2003

**Figure 4:**
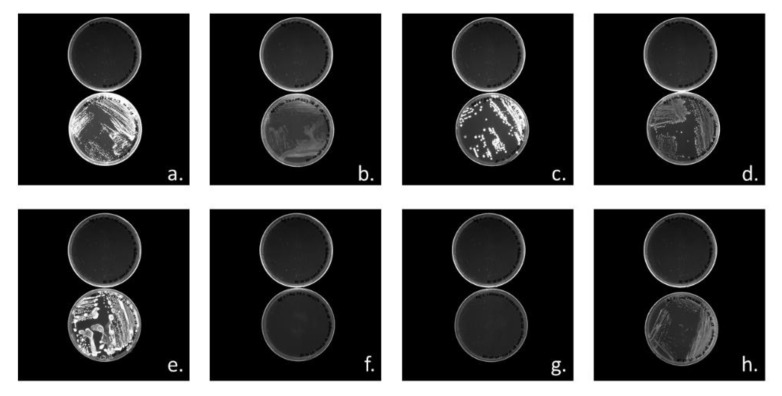
Nile Red agar plates screening with *Halomonas* sp. SF2003 using 2% (w/v) of different carbon substrates. Bacterial growth was evaluated under white light by the presence, or no, of colonies. PHA production was evaluated under Ultra-Violet light (UV-light) by fluorescence emission from colonies; positive results appear as “white” colonies showing their fluorescence. The positive control (medium without addition of carbon substrates) is the upper plate in (**a**) to Figure. (**a**). (D)-Glucose, (**b**). (D)-Fructose, (**c**). (D)-Galactose, (**d**). (D)-Mannose, (**e**). (D)-Maltose, (**f**). (D)-Melibiose, (**g**). (L)-Rhamnose, and (**h**). (D)-Sucrose. Observations under UV-lights performed with transillumination.

Visual examinations of Nile Red agar plates allow to detect colonies and bacterial growth and PHA production have been screened by detection of Nile Red fluorescence under UV-lights. *Halomonas* sp. SF2003 was able to use a majority of the tested carbohydrates as substrates for both bacterial growth and PHA accumulation: (D)-Glucose (Figure 4a), (D)-Fructose (Figure 4b), (D)-Galactose (Figure 4c), (D)-Mannose (Figure 4d), (D)-Maltose (Figure 4e) and (D)-Sucrose (Figure 4h), only (L)-Rhamnose and (D)-Melibiose were not used (Figure 4f,g and Table 2). On Nile Red agar plates, the number of colony-forming units (CFU), as well as the size of the colonies, vary from one carbohydrate to another. Qualitatively, growth of *Halomonas* sp. SF2003 seems to be more important on (D)-Glucose, (D)-Mannose, and (D)-Maltose, but comparatively, PHA production seems to be more efficient on (D)-Glucose, (D)-Galactose and (D)-Maltose, based on fluorescence intensity.

As described previously and illustrated in Table 3, *Halomonas* sp. SF2003 is able to grow in medium with (D)-Glucose, (D)-Fructose, (D)-Galactose, (D)-Mannose, (D)-Maltose and (D)-Sucrose. The strain also seems able to use these carbohydrates for PHA production in accordance with genomic analysis [13]. Indeed, the study of *Halomonas* sp. SF2003 genome highlighted the presence of various genes coding for enzymes responsible for carbohydrates assimilation such as fructose or sucrose. However, some of the tested carbohydrates have also been used by *Halomonas* sp. SF2003, despite the preliminary study of its genome only identified a part of genes required for their total assimilation (Table 3). These results suggest the interest of performing a re-examination and annotation of *Halomonas* sp. SF2003 genome but also open the door for new studies/productions using these pure carbohydrates, which can easily be found in various food or agri-food (co-)products.

The use of these carbohydrates for PHA production has already been reported in different bacterial species, including, or not, *Halomonas* species (Table 4). All these carbohydrates allow to produce P-3HB, and sometimes P-3HB-*co*-3HV, with a yield of production ranging from 0.11 g/L to 64.0 g/L of PHA.

Results of production show that the employed bioprocess (meaning strain, carbon sources, and production systems) significantly impacts production yields and composition of the polymer. There are plenty of systems that can be used; therefore, it is difficult to designate which one is the most effective. However, data described previously and in Table 4 show the importance of a deep study and judicious choice of the employed bioprocess. Data also demonstrate capacity of *Halomonas* species to use a wide variety of carbohydrates for PHA production in accordance with results obtained with *Halomonas* sp. SF2003, and are sometimes more efficient than non-halophilic strains. To complete data about *Halomonas* sp. SF2003 carbohydrates metabolisms, additional tests have been conducted on one simple sugar: fructose, galactose, and glucose or mixed with one fatty or organic acids, in the proportion 95:5% (mol/mol). Such acids have already been reported as a precursor for the biosynthesis of copolymers when simple sugars were used as the main substrate. The following acids, which are components of plants, fruits, or different industrial effluents (agri-food, chemical, cosmetic, pharmaceutical), were tested: dodecanoic, heptanoic, hexanoic, levulinic, malic, palmitic, and trans-2-pentenoic. In the same way than for screening tests with pure carbohydrates, bacterial growth has been evaluated by visual examination and PHA production by detection of fluorescence under UV-lights.

**Figure 5:**
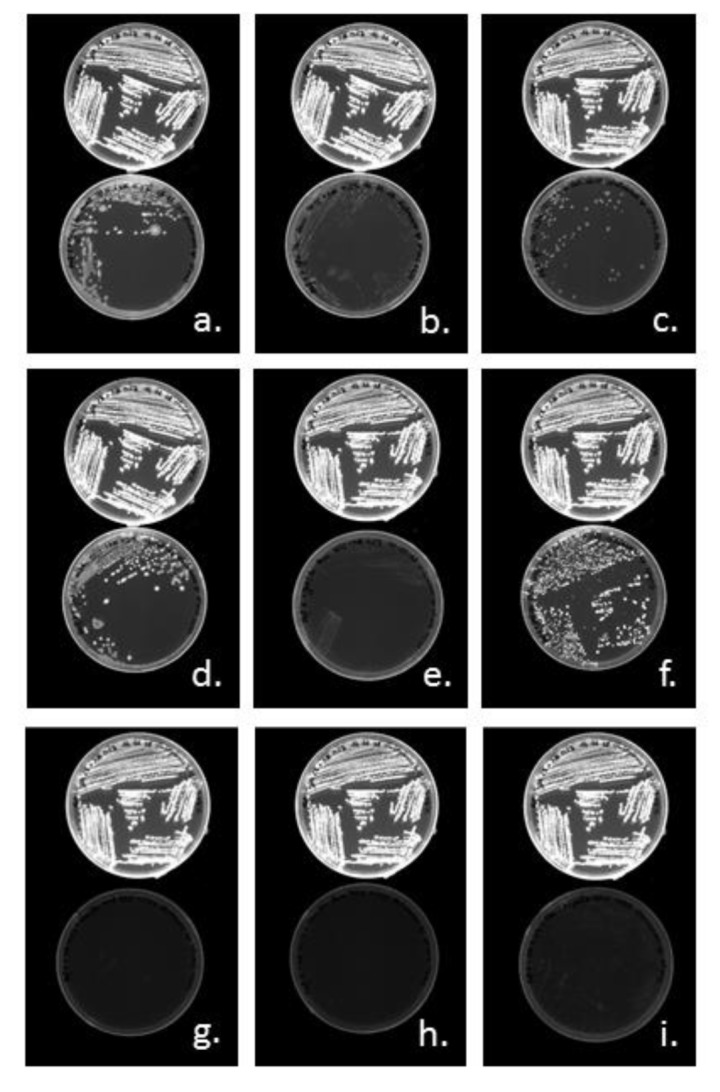
Nile Red agar plates screening with *Halomonas* sp. SF2003 using carbohydrates/acid mix with molar ratio 95/5%, final concentration 2% (w/v). Bacterial growth was evaluated under white light by the presence, or no, of colonies. PHA production was evaluated under UV-light by fluorescence emission from colonies, positive results appear as “white” colonies showing their fluorescence. Positive results appear as “white” colonies showing their fluorescence. The positive control (Glucose only) is the upper plate in (**a**–**i**). (**a**). Mix Glucose-Malic acid, (**b**). Mix Glucose-Levulinic acid, (**c**). Mix Glucose-Palmitic acid, (**d**). Mix Galactose-Malic acid, (**e**). Mix Galactose-Levulinic acid, (**f**). Mix Galactose-Palmitic acid, (**g**). Mix Fructose-Malic acid, (**h**). Mix Fructose-Levulinic acid and (**i**). Mix Fructose-Palmitic. Observations under UV-lights performed with transillumination.

*Halomonas* sp. SF2003 can grow on majority mixtures composed of glucose or galactose and organic acids except the following: glucose-dodecanoic acid and galactose-dodecanoic/heptanoic/hexanoic acids. A mixture of fructose and acids cannot be used for bacterial growth nor PHA production, whatever the acid (Table 5). This finding suggests an inhibitory effect of acids depending on the sugar used as co-substrate. Among the mixture allowing growth, only five exhibit fluorescence under UV-lights, suggesting PHA production: glucose-malic acid (Figure 5a), glucose-levulinic acid (Figure 5b), glucose-palmitic acid (Figure 5c), galactose-malic acid (Figure 5d) and galactose-palmitic acid (Figure 5f and Table 5).

According to data reported here and in the literature, it appears that numerous bacterial species, including *Halomonas* sp. SF2003, can use several pure carbohydrates for growth and also for PHA production.

This ability to exploit various carbon substrates, in addition to its capacity to grow in front of atypical/stressful conditions, make *Halomonas* sp. SF2003 a versatile strain with a high potential for biotechnological application/use [13,16]. The results of this study identify several potential carbon substrates allowing PHA production and open the door for future tests studying the exploitation of each one.

### 3.3. Study of PHA Synthases

PHA biosynthesis activity of *Halomonas* sp. SF2003 is due to the presence of genes coding for enzymes linked to PHA metabolism (i.e., *phaA*, *phaB*, *phaC1*, *phaC2,* and *phaR*). Interestingly, genes coding for acetyl-CoA acetyltransferase (also known as *β*-ketothiolase) (*phaA*), acetoacetyl-CoA reductase (*phaB*), and PHA synthases (*phaC1* and *phaC2*) are not organized in one operon but are distant from each other on *Halomonas* sp. SF2003 genome sequence. Moreover, *phaC1* and *phaC2* genes expose atypical sizes (1965 bp and 2865 bp, respectively), and conserved domain, which led to further study of both genes.

#### 3.3.1. Cloning of PHA Synthases phaC1 and phaC2 of *Halomonas* sp. SF2003

Gene *phaC1* has been amplified using PhaC1-F and PhaC1-R primers and *phaC2* gene using PhaC2-F and PhaC2-R. PCR allowed amplicons production of approximatively 2000 and 3000 bp, respectively, corresponding to *phaC1* and *phaC2* size (1965 pb and 2865 pb, respectively).

#### 3.3.2. Characterization of PHA Production by Transformant Strains PHB¯4/pBBR1-Pro_Cn_-phaC1 and PHB¯4/pBBR1-Pro_Cn_-phaC2

To evaluate the functionality of PHA synthases PhaC1 and PhaC2 of *Halomonas* sp. SF2003, screening for bacterial growth and PHA production have been performed. Likewise, with wild type *Halomonas* sp. SF2003, a total of eight carbohydrates and twenty-one mixtures, have been tested. Bacterial growth and PHA production were qualitatively checked using Nile Red agar plates technique with white light and UV-light evaluation (Supplementary data S2: Nile Red agar plates screening with PHB¯4/pBBR1-Pro_Cn_-*phaC1* using 2% (w/v) of different carbon substrates, Picture a–h; Supplementary data S2: Nile Red agar plates screening with PHB¯4/pBBR1-Pro_Cn_-*phaC2* using 2% (w/v) of different carbon substrates, Picture a–h and Table 6).

PHB¯4/pBBR1-Pro_Cn_-*phaC1* was able to exploit all pure carbohydrates and a majority of mixtures of carbohydrates/acids tested for bacterial growth (Supplementary data S2: Nile Red agar plates screening with PHB¯4/pBBR1-Pro_Cn_-*phaC1* using 2% (w/v) of different carbon substrates, Picture a–h and Table 6) except the following mixtures: glucose – heptanoic/hexanoic/trans-2-pentenoic acids, galactose – heptanoic/hexanoic/palmitic/trans-2-pentenoic (data not shown). In comparison, results obtained with PHB¯4/pBBR1-Pro_Cn_-*phaC2* are similar, for pure carbohydrates and mixtures, except for a mixture of galactose-palmitic acid for which growth was recorded (Supplementary data S3: Nile Red agar plates screening with PHB¯4/pBBR1-Pro_Cn_-*phaC2* using 2% (w/v) of different carbon substrates, Picture a–h and Table 6). Some results did not appear clearly positive and have been denoted as “±” making interpretation of substrates used and PHA production difficult.

PHA production has been detected with both transformant strains, in comparison with the mutant *C. necator* PHB¯4 (DSM 541) (data not shown), demonstrating the success of cloning experiments and functionalities of both PHA synthase genes, *phaC1* and *phaC2*. Screening tests have allowed the confirmation of correct annotation of *phaC1* and *phaC2* genes and attest to the existence of the difference between both PHA synthases of *Halomonas* sp. SF2003. Indeed, qualitative analysis of PHA accumulation, by detection of fluorescence under UV-light highlighted several differences between both transformant strains. Indeed, among all carbon substrates tested, only three seem to allow PHA accumulation in PHB¯4/pBBR1-Pro_Cn_-*phaC1* (Fructose, Glucose-dodecanoic/palmitic acid) and nine for PHB¯4/pBBR1-Pro_Cn_-*phaC2* (Fructose, Mannose, Sucrose, Glucose-dodecanoic/palmitic acids, Glucose-levulinic/malic acids, Galactose-levulic/malic acids).

These results suggested that synthase PhaC1 was less active or more selective than PhaC2. Actually, qualitatively, there were more carbon substrates (pure or in the mixture) that generated a fluorescence under UV-light when PHB¯4/pBBR1-Pro_Cn_-phaC2 was used than PHB¯4/pBBR1-Pro_Cn_-phaC1. A previous study of *Halomonas* sp. SF2003 genome and metabolisms demonstrated several differences between both synthases [13]. PhaC1 and PhaC2 had an identity of 60–70% and 65–96%, respectively, with different PHA synthases [13]. Results of both studies are in agreement with each other. Even if PhaC2 exposes some atypical characteristics (size and structure of conserved domains), it seems to be the main PHA synthase responsible for PHA biosynthesis.

#### 3.3.3. Polyhydroxyalkanoates Production in Shake Flasks

Results of screening tests demonstrated that several carbohydrates could be used for PHA production by the transformant strains. To evaluate the transformant strains, PHB¯4/pBBR1-Pro_Cn_-*phaC1*, and PHB¯4/pBBR1-Pro_Cn_-*phaC2*, the production of PHA was compared with *Halomonas* sp. SF2003 and *C. necator* H16 in glucose, fructose, and galactose. The functionality of PhaC1 and PhaC2 of *Halomonas* sp. SF2003 also can be compared based on the results of PHA production.

For *Halomonas* sp. SF2003, glucose was the favorite carbohydrate to produce PHA production (2.25 g/L) followed by galactose (1.23 g/L) and then fructose (1.02 g/L) (Table 7). Comparatively, *C. necator* H16 produces more PHA when fructose is used as the main carbon source in medium (2.25 g/L) rather than glucose (2.05 g/L). Production using galactose cannot be estimated due to low cell dry weight obtained.

Expression of pBBR1-Pro_Cn_-*phaC1* and pBBR1-Pro_Cn_-*phaC2* plasmids allow PHA accumulation in *C. necator* mutant strain PHB¯4 (a non-PHA-producing strain), further confirm the functionality of PhaC1 and PhaC2. Similar to *C. necator* H16, PHB¯4/pBBR1-Pro_Cn_-*phaC2* uses more efficiently fructose for PHA production (1.38 g/L) than glucose. Likewise, with *C. necator* H16, PHA production tests using galactose did not allow to determine production yield for both PHB¯4/pBBR1-Pro_Cn_-*phaC1* and PHB¯4/pBBR1-Pro_Cn_-*phaC2*.

It was also highlighted that galactose is more adapted for the growth of *Halomonas* sp. SF2003 than for PHA synthesis since only 39 wt% of PHA content was estimated, whereas 86 wt% of PHA content was accumulated when glucose was used (Table 7). With *C. necator* H16, fructose seems to be more exploited for bacterial growth than for PHA production, PHA content was the same as in glucose condition (71 wt%). PHB¯4/pBBR1-Pro_Cn_-*phaC1* showed a stronger growth with galactose, very close to those obtained with glucose, rather than with fructose. However, PHB¯4/pBBR1-Pro_Cn_-*phaC1* didn’t show PHA accumulation, while PHA content was quite similar using fructose or glucose as the carbon source: 33% and 30%, respectively (Table 7). Finally, PHB¯4/pBBR1-Pro_Cn_-*phaC2* uses more efficiency fructose for bacterial growth and PHA production than glucose, even if PHA contents are again quite similar (54% for fructose and 52% for glucose).

## 4. Discussion

Lipase box-like sequences are highly conserved domains which have been identified as active sites of the enzymes [31,32,33] and play a crucial role in elongation of the polymer [34] in several PHA-producing species. These domains expose similarities with those of lipase, but the difference is in the replacement of the essential active site of lipase, a serine, by a cysteine in the lipase box-like domain of PHA synthase [35], leading to renaming these sequences as PhaC box consensus sequences [34]. In this pattern, similar to lipase, Cysteine (Cys or C) represents the catalytic amino acid and is involved in a catalytic triad (C-H-D) participating, supposedly, in the elongation step of the PHA polymer [34]. The most common described pattern is Glycine-X-Cysteine-X-Glycine (G-X-C-X-G), including Glycine-Tyrosine-Cysteine-Methionine-Glycine sequence (G-Y-C-M-G) detected in *Bacillus cereus* (ATCC 14579) or *Haloferax mediterranei* (ATCC 33500), Glycine-Tyrosine-Cysteine-Leucine-Glycine sequence (G-Y-C-L-G) found in *Cupriavidus metallidurans* strain CH34 or *Halomonas boliviensis* LC1 (DSM15516), or Glycine-Alanine-Cysteine-Serine-Glycine sequence (G-A-C-S-G) in *Cupriavidus necator* strain N-1 or *Pseudomonas fulva* strain 12-X [31,34,35,36]. However, variations in amino acid composition have also been described in various bacterial species like *H. elongata* (DSM 2581) or *Halomonas* sp. KM-1 for which sequences have been described (Figure 3) [31].

In silico study of PHA synthases allowed to identify two different lipase box-like sequences: Glycine-Tyrosine-Cysteine-Leucine-Glycine (G-Y-C-L-G) for PhaC1 and Serine-Tyrosine-Cysteine-Isoleucine-Glycine (S-Y-C-I-G) for PhaC2. The G-Y-C-L-G pattern detected in PhaC1 amino acids sequence has already been reported in different halotolerant/halophiles (or not) PHA/PHB-producing strains as *C. metallidurans* strain CH34 [37] or *H. boliviensis* LC1 (DSM 15516) [38]. The second pattern, S-Y-C-I-G, founds in the PhaC2 amino acid sequence has also been reported in sequence of other halotolerant/halophiles PHA/PHB-producing strains as *Chromohalobacter salexigens* (DSM 3043) and *Halomonas* sp. KM-1 [31,39,40]. Both lipase box-like sequences of *Halomonas* sp. SF2003 PHA synthases have a tyrosine, a cysteine, and a glycine (Y-S-G) suggesting that these residues can potentially have a crucial role in the catalytic activity of the enzymes.

These results confirm the distinction of both enzymes, additionally to their size and location in the genome [13]. The differences between both PHA synthases of *Halomonas* sp. SF2003 could generate a difference in catalytic activity and potentially, in the end, impact yield of polymer production. Further research must be performed to elucidate impact of each pattern on enzymes substrates specificity and selectivity and also to validate the identification of catalytic core of both. To complete data, structural study of enzymes exploiting X-ray crystallography and/or molecular biology could be performed to confirm, or not, that the identified lipase box-like sequences play a key role in the synthesis of PHA by *Halomonas* sp. SF2003.

Alterations/modifications of PHA synthase sequences, using molecular biology, will lead to change proteins tertiary structures and potentially synthesis activity. Indeed, other studies have already been performed to elucidate the tertiary structure of different PHA synthases and to identify active sites. For example, Ilham et al. (2014) have studied PHA synthases of *Halomonas* sp. O-1 by performing site-directed mutagenesis on different residues and studying the production of the strain. They determined that appropriated changes can, positively or negatively, affect synthesis activity, bacterial growth, or molecular weight of polymers. Th substitution of alanine for Cys329 or Cys331 in *Halomonas* sp. O-1 or *H. elongata* DSM 2581 PHA synthase sequence leads to a total inhibition of PHA synthesis while substituting glycine for serine impacts polymer molecular weight. These results allowed identification of catalytic sites in enzymes and to imagine modifications in strain genes to enhance production [31]. Same kind of experiments would be one of the prospects to deeply characterize PHA synthases, PhaC1 and PhaC2, of *Halomonas* sp. SF2003. Futhermore, studies exploiting X-ray crystallography will allow to apprehend structure of the catalytic site and to confirm the role of each residue [35]. Similar studies have already been performed and reported with different species, such as *Chromobacterium* sp. USM2 [41], *C. necator* [42] or *Pseudomonas* sp. 61–3 [43].

To investigate the ability of *Halomonas* sp. SF2003 to produce different PHA, various carbon substrates and mix have been screened for growth and biopolymer accumulation using the Nile Red agar plates technique. Nile Red is a fluorescent stain of intracellular lipids, and hydrophobic domain, frequently used to detect PHA [44]. Indeed, the Nile Red represents an easy and fast detection tool for PHA biosynthesis using various technic as agar plates or epifluorescence microscopy [11,45,46,47]. Work has been mainly focused on “pure” carbon substrates, like carbohydrates, for a better understanding of PHA synthase activity and specificity. Eight pure carbohydrates, including five monosaccharides (glucose, fructose, galactose, rhamnose, mannose) and three disaccharides (maltose, melibiose, sucrose) found in food or natural (co-) products, including fruits, vegetables, milk or red algae, have been tested based on data available in the literature and results of previous studies on *Halomonas* sp. SF2003 [12,13,16,31,48]. The results obtained with *Halomonas* sp. SF2003 confirmed the substrate versatility of this species for both growth and PHA production. Among the tested carbohydrates, positive results have been recorded with glucose, fructose, galactose, mannose, maltose, and sucrose.

Only one carbohydrate in a (L) configuration has been tested: (L)-Rhamnose, and it does not allow both bacterial growth and PHA accumulation by *Halomonas* sp. SF2003. The inability to use (L)-Rhamnose for bacterial growth or PHA production by *Halomonas* sp. SF2003 suggests a lack in part or in totality, of required metabolic tools. This hypothesis is in accordance with the results of a previous study, which showed the presence of only a few genes responsible for rhamnose (II) degradation in the *Halomonas* sp. SF2003 genome (Table 3) [13]. At this time and to our knowledge, there are not yet reports of PHA production with (L)-rhamnose using *Halomonas* species. In contrast, ability to use this sugar as carbon source and substrate for PHA production is variable since *Halomonas* species as *H. cupida*, *H. elongata*, or *H. maura* [49,50] or other species as *C. necator* or *Pseudomonas oleovorans* [51] are capable of doing so while *Halomonas* species as *H. aquamarina*, *H. hamiltonii* (DSM 21196T) or *H. subterranea* (JCM 14608T) are not, in accordance to results obtained with *Halomonas* sp. SF2003. (D)-Melibiose was the second one carbohydrate, which does not allow bacterial growth. The results of screening tests performed suggest that *Halomonas* sp. SF2003 does not or only possesses a part of enzymes required for (D)-Melibiose degradation contrary to results suggested by our previous in silico study [13]. A limited number of studies deal with use of (D)-Melibiose for bacterial growth and only sometimes for PHA production as *Burkholderia sacchari* sp. nov. [17]. *H. cupida* is also able to use (D)-Melibiose for its growth [50] like *Bacillus* sp. (Strain SKM11) [52], *Bacillus subtilis* (Strain PHA 012), *Aeromonas* sp. (Strain PHA 046) or *Alcaligenes* sp. (Strain PHA 047) [53]. Comparatively, and similarly to *Halomonas* sp. SF2003, numerous PHA-producing species have also been reported for their disability to exploit (D)-Melibiose for growth as *Pandoraea* sp. (Strain MA 03) [54], or *Bacillus cereus* (Strain FC11) [55].

In the case of *Halomonas* sp. SF2003, and based on the results of the screening tests, it makes more sense to use (D)-Glucose, (D)-Galactose, and (D)-Maltose, which show to qualitatively allow a stronger PHA production. To complete these results, it is necessary to test a mixture of these sugars with different ratios of each of them in order to evaluate if PHA production is stronger when exploiting them alone or combined. Moreover, the evaluation of PHA production with a mixture of carbohydrates will allow the identification of potential co-products usable with *Halomonas* sp. SF2003. Indeed, bacterial growth and PHA production of various strains using pure carbohydrates is frequently tested and well reported [17,23]. However, because of their high cost, their use at the industrial scale cannot be reasonably considered and exploitation of by-products is privileged [6]. For example, the production of PHA is rarely tested with “pure” galactose but rather using products constituted by itself such as lactose sources (lactose or cheese whey and milk) or in its polymeric form such as agar in red algae in order to promote the use of various co-products. Indeed, PHA productions have successfully been performed with *Haloferax mediterranei* and *Pseudomonas hydrogenovora* on whey lactose [56,57,58], or with *Bacillus megaterium* [59] using acid-treated red algae. Actually, there is an important number of studies using this group of carbon substrates. However, without more precise analyzes, it is difficult to know which carbohydrates are preferentially used for PHA accumulation. Moreover, the use of these products in their original forms by bacterial strains is difficult, and consequently, some pre-treatments (hydrolysis) are required, sometimes leading to an increase in the cost and time of production [5]. Similar experiments of PHA production have also been performed using (pre-treated) co-products composed of mannose, such as spent coffee ground [60], sugar maple hemicellulosic hydrolysate [61] or ensiled grass press juice [62]. A majority of pure carbohydrates tested for bacterial growth and PHA production in this study can be found in industrial or natural products (Table 3), allowing to test the assimilation/exploitation of different (co-)products by *Halomonas* sp. SF2003.

Additional tests have been conducted on one simple sugar: fructose, galactose, and glucose or mixed with one fatty or organic acids to complete data about *Halomonas* sp. SF2003 carbohydrates metabolisms and to identify new potential carbon sources. Based on the results of the screening tests performed with *Halomonas* sp. SF2003, and using a mix of carbohydrates and acids, production tests might be achieved. In fact, a mix exposing positive results is composed of carbohydrates and acids, which are easily found in various natural products or by-products [63,64].

Levulinic, malic, and palmitic acids can easily be found in plant co-products and have already been tested in a mix with different carbon substrates for PHA production by different bacterial strains. Levulinic acid has been employed in a mix with xylose to perform PHA production with *Burkholderia cepacia* [65] or combined to glucose/fructose with *C. necator* [66]. Quantities of acid employed vary to those tested here and lead to the production of P-3HB-*co*-3HV up to 2.40 g/L with *B. cepacia* [65] and P-3HB synthesis up to 2.41 g/L for *C. necator* [66]. Alongside, previous studies for assimilation of levulinic acid has also been evaluated with *Halomonas hydrothermalis* using seaweed-derived crude levulinic acid and lead to accumulation of P-3HB-*co*-3-HV up to 1.07 g/L [63]. The second acid, malic acid, has been used as co-substrates for PHA production with different bacterial species, such as *B. sacchari,* which accumulates P-3HB up to 2.80 g/L from mix of glucose and malic acid [67]. By-products composed of malic acid from fruit pomace have successfully been exploited by *Pseudomonas resinovorans* for poly-3-hydroxyhexanoates-*co*-3-hydroxyoctanoate-*co*-3-hydroxydecanoate-*co*-3-hydroxydodecanoate-*co*-3-hydroxytetradecenoate (P-3HHx-*co*-3HO-*co*-3HD-*co*-3HDD-*co*-3HTD) production reaching 1.27 g/L [68]. Other papers reported that the addition of malic acid in the production medium of *Methylobacterium trichosporium* can promote the production of P-3HB up to 1.94 g/L [69]. Finally, palmitic acid is also frequently exploited for PHA production and with various bacterial species. Cruz et al. have tested several by-products and wastes as carbon substrates for PHA production, including olive oil, cooking oil, or biodiesel fatty acids by-products. All these products contain more or less important quantities of various fatty acids including palmitic acid. PHA production has been estimated with different species as *Pseudomonas citronellolis*, *P. oleovorans*, *P. resinovorans*, *C. necator* H16 and *C. necator* NRRL B-4383 and demonstrated viability of used wastes and by-products [70]. Another study exploited oil of spent coffee ground, which contains palmitic acid, for P-3HB production with *C. necator* H16 and led to productions reaching up 10 g/L [71]. Additionally, to *Pseudomonas* and *Cupriavidus* species, tests have been conducted on *Burkholderia* sp. USM (JCM15050) to evaluate the exploitation of representative quantities of palmitic acid, alone or in different by-products. The results of this study demonstrated a higher production of P-3HB, up to 1.25 g/L, using palm oil products rather than pure palmitic acid (0.14 g/L of P-3HB) [72].

Consequently, previous tests could also be completed using more or less important different carbohydrates/acids ratio, as performed in different studies. However, the Nile Red agar plates’ tests are only used as screening tool and must be completed with production tests to evaluate the impact of each mix or pure carbon substrates on production yield and polymer composition.

Furthermore, additional tests exploiting different by-products derivatives of dairy, waste treatment or agri-food industries might be performed in order to evaluate viability of exploiting these co-products and to optimize PHA production by *Halomonas* sp. SF2003. This kind of production has already been done with different bacterial species, including *Halomonas* species, as well as other ones, as described previously. Indeed, Pernicova et al. 2019, have studied the viability of several *Halomonas* strains to produce PHA from waste cooking oil. They demonstrated that *Halomonas hydrothermalis* exposes the highest production yield (0.38 g/L) but also the influence of NaCl concentration on production [73]. These results demonstrate that the production medium must be wisely studied and elaborated.

The efficiency of two transformed strains harboring *phaC1* or *phaC2* genes was estimated and compared to those of the wide bacteria through lab-scale production. Data of this study confirm their functionality and existence of differences between them, including their size, sequences, location on genome, and capability of production of PHA. Indeed, PhaC2 exhibited greater PHA production capability as PHB¯4/pBBR1-Pro_Cn_-*phaC2* accumulated more PHA in comparison to that of PHB¯4/pBBR1-Pro_Cn_-*phaC1* under the exact same host, plasmid, and culture conditions. This is in accordance with previous results demonstrating a higher percent of the identity of PhaC2 with PHA synthases of other bacterial species than PhaC1 [13]. To confirm this result, PHA production tests must be conducted, and new genetic constructions could be tested. These tests will allow the definite evaluation of the functionality of PHA synthases. Moreover, in this study, a pBBR1MCS-2 plasmid with *C. necator* H16 promoter was used. This construction, could be responsible, in part, for the weak activity of PhaC1. Indeed, in *Halomonas* sp. SF2003 genome PHA biosynthesis genes expose an atypical distribution [13], so PHA synthases expression of PhaC1 and PhaC2 was tested separately. However, it could be possible that PhaC1 specifically requires proteins (PhaC2, PhaA, PhaB) or promoter of *Halomonas* sp. SF2003 metabolisms to ensure polymer synthesis despite that it has been identified to belong to class I of synthase (meaning that PhaC are constituted of only one subunit and does not require any additional protein to be active). To confirm this hypothesis, several different constructions might be designed using *Halomonas* sp. SF2003 promoter and PHA biosynthesis genes simultaneously, and evaluated for PHA production [35,74,75,76]. Among all the different constructions which could be tested, plasmid harboring both *phaC1* and *phaC2* genes, together, must be designed. This construction will allow the control of the influence of each one on the other and to check if PHA synthase PhaC1 requires PhaC2 to be active. Testing different constructions will allow a better understanding of genes activity and to identify the best combination to optimize production.

Finally, both transformant strains showed lower efficiency of PHA production than the wild type strain *C. necator* H16. Lower PHA production could be due to the weaker acetyl-CoA C-acyltransferase and *β*-ketothiolase activities, as already described by Mifune et al. 2008 [77]. PhaCs of *Halomonas* sp. SF2003 can also expose a lower activity than PhaC of *C. necator* H16.

The composition of production medium and production parameters used for these tests can also be responsible in part of the low production yields. Indeed, the same medium and parameters have been used for all the strains. However, *Halomonas* sp. SF2003 and *C. necator*’s wild and transformant strains do not exhibit the same origin and metabolisms. Consequently, new production medium and different production parameters must be tested. Following production of each strain in different conditions will allow more precise understanding of the activity of each strain/PHA synthase and to adjust more precisely the production step.

## 5. Conclusions

This study has demonstrated the functionality of both PHA synthases, PhaC1, and PhaC2, confirming annotation of *Halomonas* sp. SF2003 genome performed in our in-silico study. Performed screening tests allowed the identification of several carbon substrates, pure carbohydrates or a mix of sugars and acids, potentially usable for PHA production by *Halomonas* sp. SF2003. Substrate versatility of this bacterium opens the door for new tests in order to optimize production and also confirm its high biotechnological potential. Preliminary biosynthesis tests expose a better PHA production using glucose with *Halomonas* sp. SF2003 while *C. necator* wild type and transformant strain preferably exploit fructose. Results also highlighted higher PHA biosynthesis ability of PHA synthase PhaC2 as compared to PhaC1. These results open the door to future research in order to overexpress the *phaC2* gene of *Halomonas* sp. SF2003 to increase the yield of production of the strain. Additional research, such as kinetics of bacterial growth and PHA production, should optimize production step.

## Figures and Tables

**Table 1:** List of plasmids and bacterial strains used in this study. DSMZ: Leibiz Institute DSMZ-German Collection of Microorganisms and Cell Cultures. Km^R^: Resistance to kanamycin, RIDL: Research Institute Dupuy de Lôme, UBS: Université de Bretagne Sud.

Strain or Plasmid	Characteristics	Origin
***Halomonas* strain**		
*Halomonas* sp. SF2003 ID CNCM-I-4786	Wild type PHA-producing strain	Sea of Iroise (France), RIDL Collection, UBS
***Cupriavidus necator* strains**		
H16 (DSM 428)	Wild type PHA-producing strain	DSMZ Collection
PHB¯4 (DSM 541)	Mutant non-PHA-producing strain	DSMZ Collection
***Escherichia coli* strains**		
*E. cloni*^®^ 10G	Competent cells	Lucigen
S17-1	Strain for conjugative transfer of plasmid to *C. necator* PHB¯4	Simon et al. 1983 [14]
**Plasmids**		
pBBR1-Pro_Cn_	pBBR1MCS-2 derivatives with *phaC1* promoter from *C. necator*. Km^R^.	Foong et al. 2014 [15]
pBBR1-Pro_Cn_-*phaC1*	pBBR1MCS-2 derivatives with *phaC1* promoter from *C. necator* and *phaC1* of *Halomonas* sp. SF2003. Km^R^.	This study
pBBR1-Pro_Cn_-*phaC2*	pBBR1MCS-2 derivatives with *phaC1* promoter from *C. necator* and *phaC2* of *Halomonas* sp. SF2003. Km^R^.	This study
**Transformants**		
PHB¯4/pBBR1-Pro_Cn_-*phaC1*	Transformant strain with pBBR1MCS-2 plasmid expressing *phaC1* of *Halomonas* sp. SF2003. Km^R^.	This study
PHB¯4/pBBR1-Pro_Cn_-*phaC2*	Transformant strain with pBBR1MCS-2 plasmid expressing *phaC2* of *Halomonas* sp. SF2003. Km^R^.	This study

**Table 2:** Growth and PHA accumulation in *Halomonas* sp. SF2003 using different carbon sources.

Carbon Source	Growth	PHA Accumulation
(D)-Glucose	+	+
(D)-Fructose	+	+
(D)-Galactose	+	+
(D)-Mannose	+	+
(D)-Maltose	+	+
(D)-Melibiose	-	-
(L)-Rhamnose	-	-
(D)-Sucrose	+	+

**Table 3:** Listing of common carbohydrates used for PHA production.

Carbohydrates	Origin	Identification of Pathway for Assimilation
Fructose *	Fruits, Honey	Total
Galactose *	Milk, Honey, Red algae	Partial
Glucose *	Food, Metabolism of living organisms	Partial
Lactose	Dairy products	Total
Maltose *	Starch degradation (barley)	n.i
Mannose *	Fruits, Plants, Mannitol	n.i
Melibiose *	Plants, Fruits	Total
Ribose	RNA	Partial
Rhamnose *	Plants	Partial
Sucrose *	Plants	Total
Xylose	Plants	Partial

* Tested in this study for PHA accumulation, n.i: not identified in the *Halomonas* sp. SF2003 genome yet.

**Table 4:** Listing (not exhaustive) of various bacterial strains using the different tested carbohydrates for PHA production.

Carbohydrates	Bacterial Strains/Species	References
Melibiose	*Burkholderia sacchari* sp. nov.	[17]
Rhamnose	*C. necator*, *P. oleovorans*	[17]
Glucose	*Bacillus cereus* UW85, ***Halomonas* sp. TD01**, *Halomonas profundus*, *Halomonas* sp. SF2003	[18,19,20]
Fructose	*Bacillus aryabhattai* PHB10, *C. necator*, *Halomonas* TD08,*Halomonas* sp. SF2003, *H. halophila*, ***H. organivorans***, *H. salina*	[21,22,23]
Sucrose	*Azotobacter vinelandii*, ***Burkholderia sacchari* DSM 17165**, *C. necator*, *Natrinema* sp. 5TL6	[6,24,25,26]
Galactose	***Halomonas halophila***, *H. salina*, *Halomonas* sp. SF2003	[23]
Mannose	*Halomonas halophila*, ***H. organivorans***, *H. salina*	[23,27]
Maltose	*B. aryabhattai* PHB10, ***Halomonas* sp. TD08**, *H. boliviensis* LC1 and *H. campisalis*	[17,21,28,29,30]

Based on data of Verlinden et al. 2007, and completed with data from other studies. Strains in bold expose the highest PHA concentrations.

**Table 5:** Growth and PHA accumulation in *Halomonas* sp. SF2003 using a different mixture of carbohydrates and acids.

Carbon Source		Growth	PHA Accumulation
Glucose	Dodecanoic acid	-	-
	Heptanoic acid	+	-
	Hexanoic acid	+	-
	Levulinic acid	+	±
	Malic acid	+	+
	Palmitic acid	+	-
	Trans-2-pentenoic acid	+	-
Galactose	Dodecanoic acid	-	-
	Heptanoic acid	-	-
	Hexanoic acid	-	-
	Levulinic acid	+	-
	Malic acid	+	+
	Palmitic acid	+	±
	Trans-2-pentenoic acid	+	-
Fructose	Dodecanoic acid	±	±
	Heptanoic acid	-	-
	Hexanoic acid	-	-
	Levulinic acid	-	-
	Malic acid	-	-
	Palmitic acid	-	-
	Trans-2-pentenoic acid	-	-

**Table 6:** Growth and PHA accumulation in transformant strains PHB¯4/pBBR1-Pro_Cn_-*phaC1* and PHB¯4/pBBR1-Pro_Cn_-*phaC2* using different carbon sources. Legend for growth and PHA accumulation +/−: Positive/Negative.

Carbon Source		PHB¯4/ pBBR1-Pro_Cn_-*phaC1*		PHB¯4/ pBBR1-Pro_Cn_-*phaC2*	
		Growth	PHA Accumulation	Growth	PHA Accumulation
	(D)-Fructose	+	±	+	+
	(D)-Galactose	+	−	+	−
	(D)-Glucose	+	−	+	−
	(D)-Maltose	+	−	+	−
	(D)-Mannose	+	−	+	±
	(D)-Melibiose	+	−	+	−
	(L)-Rhamnose	+	−	+	−
	(D)-Sucrose	+	−	+	±
Glucose +	Dodecanoic acid	+	±	+	+
	Heptanoic acid	−	−	−	−
	Hexanoic acid	−	−	−	−
	Levulinic acid	+	−	+	±
	Malic acid	+	−	+	±
	Palmitic acid	+	±	+	+
	Trans-2-pentenoic acid	−	−	−	−
Galactose +	Dodecanoic acid	+	−	+	−
	Heptanoic acid	−	−	−	−
	Hexanoic acid	−	−	−	−
	Levulinic acid	±	−	+	±
	Malic acid	+	−	+	±
	Palmitic acid	−	−	+	−
	Trans-2-pentenoic acid	−	−	−	−
Fructose +	Dodecanoic acid	−	−	−	−
	Heptanoic acid	−	−	−	−
	Hexanoic acid	−	−	−	−
	Levulinic acid	−	−	−	−
	Malic acid	−	−	−	−
	Palmitic acid	−	−	−	−
	Trans-2-pentenoic acid	−	−	−	−

**Table 7:** Comparative PHA productions in shake flasks with glucose, fructose or galactose. Productions conducted in triplicate in 250 mL shake flasks containing 50 mL of medium with 2% (w/v) of carbon sources.

Strain	Carbon Source	Dry cell Weight (g/L)	PHA (g/L)	PHA Content (wt.%)
*Halomonas* sp. SF2003	Glucose	2.63	2.25	86
*C. necator* H16		2.89	2.05	71
PHB¯4/pBBR1-Pro_Cn_-*phaC1*		1.05	0.32	30
PHB¯4/pBBR1-Pro_Cn_-*phaC2*		2.63	1.38	52
*Halomonas* sp. SF2003	Fructose	2.63	1.02	39
*C. necator* H16		3.16	2.25	71
PHB¯4/pBBR1-Pro_Cn_-*phaC1*		0.79	0.26	33
PHB¯4/pBBR1-Pro_Cn_-*phaC2*		3.42	1.83	54
*Halomonas* sp. SF2003	Galactose	3.16	1.23	39
*C. necator* H16		0.79	N.D	N.D
PHB¯4/pBBR1-Pro_Cn_-*phaC1*		1.06	N.D	N.D
PHB¯4/pBBR1-Pro_Cn_-*phaC2*		0.79	N.D	N.D

N.D: Not determined.

## Supplementary Materials

The following are available online at https://www.mdpi.com/2306-5354/7/1/29/s1. Supplementary data S1: Accession numbers of PhaC amino acids sequences on National Center for Biotechnology Information (NCBI) database. Supplementary data S2: Nile Red agar plates screening with PHB¯4/pBBR1-Pro_Cn_-phaC1 using 2% (w/v) of different carbon substrates. Supplementary data S3: Nile Red agar plates screening with PHB¯4/pBBR1-ProCn-phaC2 using 2% (w/v) of different carbon substrates.
