# Supplementary material for: PHA Production and PHA Synthases of the Halophilic Bacterium Halomonas sp. SF2003
Source: Bioengineering (Basel). 2020 Mar 20;7(1):29. doi: 10.3390/bioengineering7010029 (PMC7175313; doi:10.3390/bioengineering7010029)
Supplement: Supplementary file 1 [file bioengineering-07-00029-s001.zip › bioengineering-706717-supplementary/Supplementary data 1 Accession numbers of PhaC amino acids sequences..docx]

| **Strains** | **Accession number of PhaC amino acids sequences** |
| --- | --- |
| *Bacillus anthracis* strain *Ames* | AAP25282.1 |
| *Bacillus cereus* (ATCC 14579) | EEL94716.1 |
| *Bacillus megaterium* (ATCC 14581) | QCY24219.1 |
| *Haloferax mediterranei* (ATCC 33500) | QCQ77213.1 |
| *Haloplanus* sp. CBA112 | WP_114605610.1 |
|  |  |
| *Alcaligenes faecalis* strain ZD02 | ALO37566.1 |
| *Bradyrhizobium diazoefficiens* strain USDA 110 | AND91204.1 |
| *Cupriavidus metallidurans* strain CH34 | ABF11981.1 |
| *Halomonas boliviensis* LC1 RS14850 (DSM 15516) | OZT74774.1 |
| *Halomonas boliviensis* LC1 RS16055 (DSM 15516) | OZT72538.1 |
| *Halomonas* sp. SF2003 (PhaC1) | WP_107334585.1 |
| *Legionella pneumophila* subsp. *pneumophila* (str. Philadelphia 1) | AAU27183.1 |
| *Ralstonia solanacearum* strain CFBP2957 | YP_003748213.1 |
|  |  |
| *Cupriavidus necator* strain N-1 | AEI77182.1 |
| *Pseudomonas cremoricolorata* strain ND07 | WP_038411120.1 |
| *Pseudomonas fulva* strain 12-X | AEF20393.1 |
| *Pseudomonas sp. (*ATCC 13867) | WP_015475174.1 |
|  |  |
| *Halomonas boliviensis* LC1 (DSM 15516) | EHJ92371.1 |
| *Halomonas campaniensis* LS21 | WP_038482670.1 |
| *Halomonas stevensii* S18214 (DSM 21198) | WP_026001773.1 |
| *Halomonas* sp. GAFJ-1 | WP_009096905.1 |
|  |  |
| *Halomonas* sp. SF2003 (PhaC2) | AVV33292.1 |
