# Supplementary material for: PHA Production and PHA Synthases of the Halophilic Bacterium Halomonas sp. SF2003
Source: Bioengineering (Basel). 2020 Mar 20;7(1):29. doi: 10.3390/bioengineering7010029 (PMC7175313; doi:10.3390/bioengineering7010029)
Supplement: Supplementary file 1 [file bioengineering-07-00029-s001.zip › bioengineering-706717-supplementary/Supplementary data captions.docx]

**Supplementary data 1: Accession numbers of PhaC amino acids sequences on National Center for Biotechnology Information (NCBI) database.**

**Supplementary data 2: Nile Red agar plates screening with PHB¯4/pBBR1-Pro_Cn_-phaC1 using 2% (w/v) of different carbon substrates.** Bacterial growth was evaluated under white light by presence, or no, of colonies. PHA production was evaluated under UV-light by fluorescence emission from colonies, positive results appear as “white” colonies showing their fluorescence. Positive results appear as “white” colonies showing their fluorescence. The positive control (Fructose only) is the upper plate on Figure 6a to Figure 6h. a. Control negative, b. Glucose, c. Galactose, d. Mannose, e. Maltose, f. Melibiose, g. Rhamnose and h. Sucrose. Observations performed under UV-lights with transillumination.

**Supplementary data 3: Nile Red agar plates screening with PHB¯4/pBBR1-Pro_Cn_-phaC2 using 2% (w/v) of different carbon substrates.** Bacterial growth was evaluated under white light by presence, or no, of colonies. PHA production was evaluated under UV-light by fluorescence emission from colonies, positive results appear as “white” colonies showing their fluorescence.Positive results appear as “white” colonies showing their fluorescence. The positive control (Fructose only) is the upper plate on Figure 6a to Figure 6h. a. Control negative, b. Glucose, c. Galactose, d. Mannose, e. Maltose, f. Melibiose, g. Rhamnose and h. Sucrose. Observations performed under UV-lights with transillumination. The positive control (Fructose only) is the upper plate on Figure 7a to Figure 7h. a. Control negative, b. Glucose, c. Galactose, d. Mannose, e. Maltose, f. Melibiose, g. Rhamnose and h. Sucrose. Observations performed under UV-lights with transillumination
