# Supplementary figures and images for: PHA Production and PHA Synthases of the Halophilic Bacterium Halomonas sp. SF2003
Source: Bioengineering (Basel). 2020 Mar 20;7(1):29. doi: 10.3390/bioengineering7010029 (PMC7175313; doi:10.3390/bioengineering7010029)

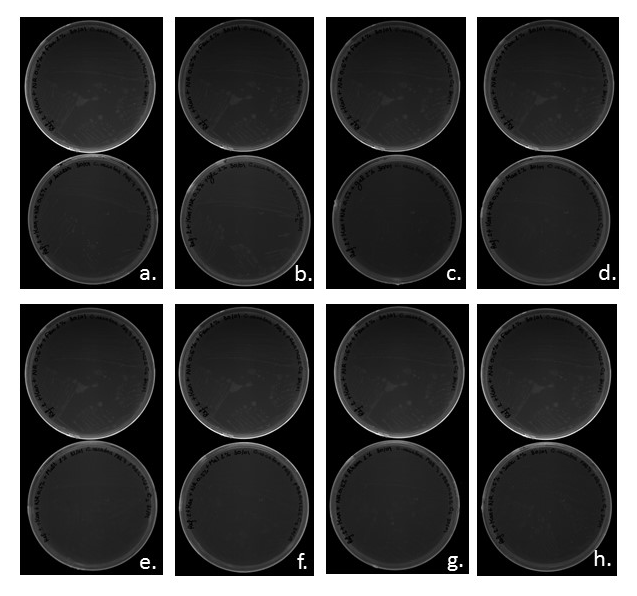

Supplement: Supplementary file 1 [file bioengineering-07-00029-s001.zip › bioengineering-706717-supplementary/Supplementary data 2 Nile Red agar plates screening with PHB_pBBR1-ProCn-phaC1 using 2% (weight volume) of different carbon substrates..tif]

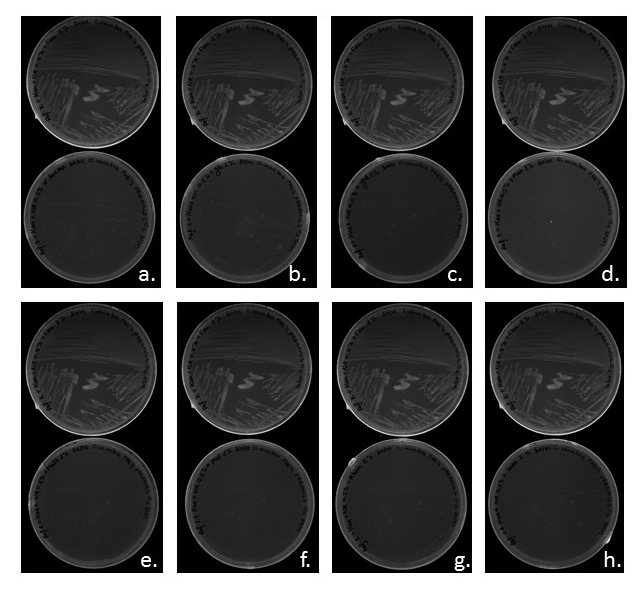

Supplement: Supplementary file 1 [file bioengineering-07-00029-s001.zip › bioengineering-706717-supplementary/Supplementary data 3 Nile Red agar plates screening with PHB_pBBR1-ProCn-phaC2 using 2% (weight volume) of different carbon substrates..tif]
